# Transcatheter arterial embolization for intercostal arterio-esophageal fistula in esophageal cancer

**DOI:** 10.1186/s40792-017-0345-8

**Published:** 2017-05-16

**Authors:** Tetsuya Tajima, Shigeo Haruki, Shinsuke Usui, Koji Ito, Akiyo Matsumoto, Akiyuki Matsuhisa, Noriaki Takiguchi

**Affiliations:** 1 Department of Surgery, Tsuchiura Kyodo General Hospital, 4-1-1 Ootsuno, Tsuchiura, Ibaraki 300-0028 Japan; 2 Department of Radiology, Saitama Cancer Center, 780 Komuro, Inamachi, Kitaadachi-gun, Saitama, 362-0806 Japan

**Keywords:** Esophageal cancer, Arterio-esophageal fistula, Intercostal artery, Transcatheter arterial embolization

## Abstract

**Background:**

While esophageal fistula formation in the adjacent organs is associated with high rates of morbidity and mortality, the management of non-aortic arterio-esophageal fistula has not been frequently reported.

**Case presentation:**

A 69-year-old Japanese man who had undergone definitive chemoradiotherapy for esophageal cancer was admitted to our hospital with hematemesis. He was diagnosed with mediastinal abscess caused by esophageal perforation, and esophageal bypass surgery was performed. After 3 days, he presented with fatal hemoptysis. As angiography revealed an intercostal artery pseudoaneurysm, transcatheter arterial embolization was performed.

**Conclusions:**

When patients with esophageal cancer, especially those with a history of radiotherapy and/or mediastinitis, present with hematemesis and/or hemoptysis, the possibility of non-aortic arterio-esophageal fistula should be considered. Transcatheter arterial embolization is an effective treatment for non-aortic arterio-esophageal fistula.

## Background

Esophageal fistula formation in the adjacent organs is associated with high rates of morbidity and mortality [1]. Although patients with esophageal cancer may suffer lethal bleeding due to aortic arterio-esophageal fistula (AEF), the management of non-aortic AEF has not been frequently reported.

We experienced a case of intercostal AEF after chemoradiotherapy (CRT) for esophageal cancer that was treated with transcatheter arterial embolization (TAE). To our knowledge, this is an extremely rare case of bleeding in esophageal cancer. We herein report the details and provide a review of the pertinent literature.

## Case presentation

**Fig. 1:**
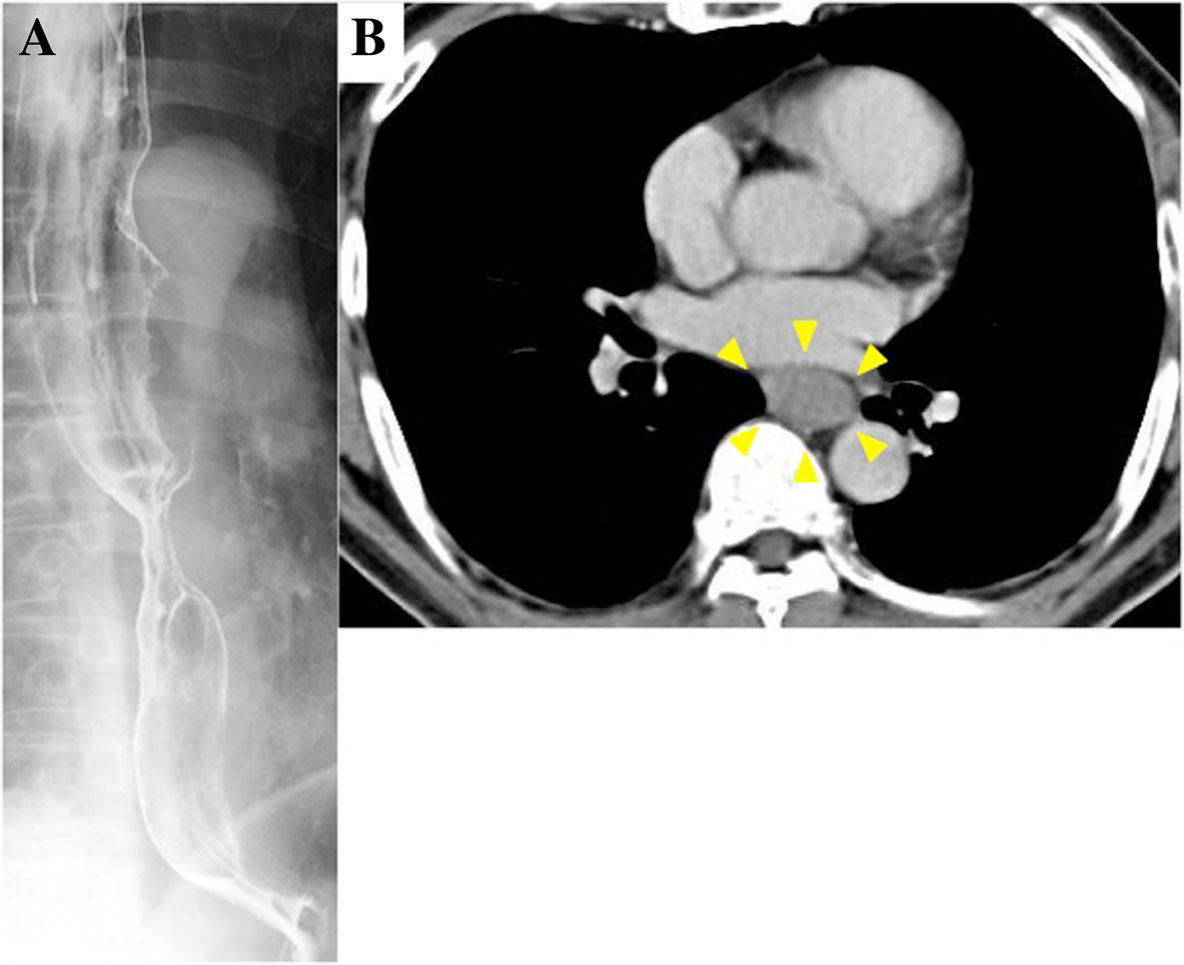
Before chemoradiotherapy. **a** Upper gastrointestinal series revealed subcircumferential esophageal stenosis at the middle thoracic esophagus. **b** Enhanced computed tomography revealed a tumor (*arrowhead*) without invasion into the surrounding tissue located at the middle thoracic esophagus

**Fig. 2:**
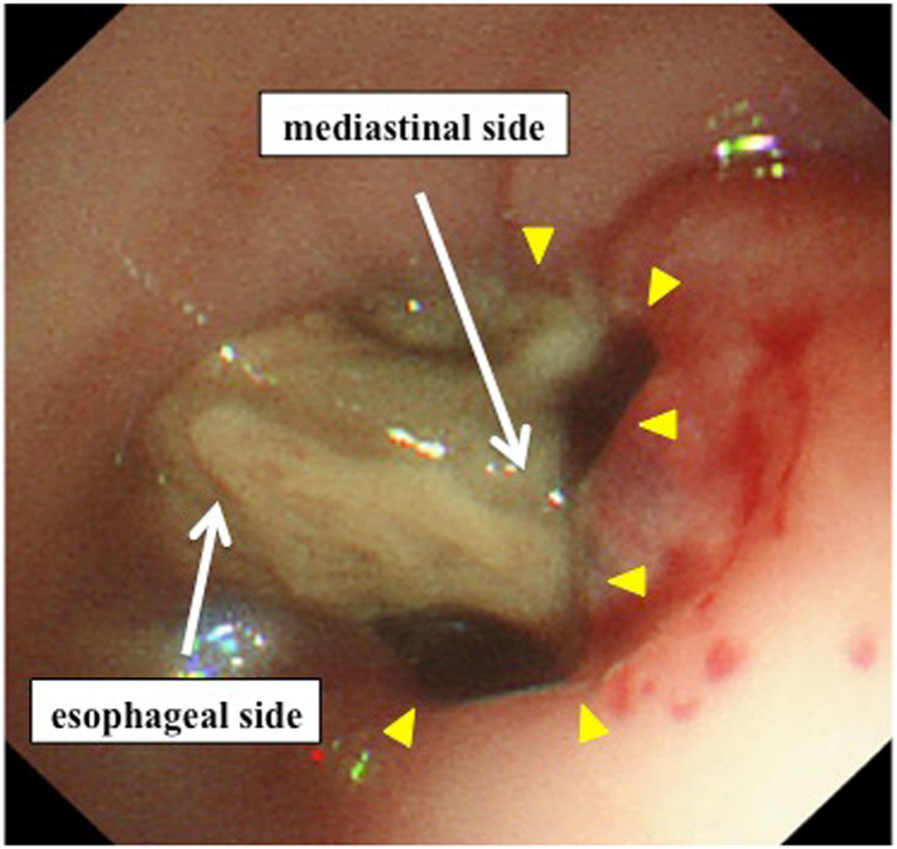
Endoscopy revealed perforation (*arrowhead*) on the right wall of the middle thoracic esophagus 32–35 cm distant from the incisors. The source of bleeding was not detected. Necrotic tissue was found obstructing the lumen of the esophagus

A 69-year-old Japanese man was admitted to our hospital with hematemesis. Five months earlier, the patient had been diagnosed with Stage IIIA (cT3N1M0) thoracic esophageal squamous cell carcinoma according to the Union for International Cancer Control, seventh edition, at the previous hospital (Fig. 1). He underwent definitive CRT, consisting of continuous infusion of 5-fluorouracil 700 mg/m^2^/day for the first 4 days of Weeks 1 and 5 and cisplatin 70 mg/m^2^ on Days 1 and 29 concurrent with external-beam radiation to a total dose of 60 Gy in 30 fractions. Endoscopy 3 months after beginning CRT revealed tumor reduction as a partial response. He had experienced back pain 2 weeks before being admitted to our facility. On admission, his vital signs were within normal limits, except for a body temperature of 37.8 °C. The laboratory data were as follows: white blood cell count 6.7 × 10^9^ /L, hemoglobin 8.0 g/dL, total protein 5.7 g/dL, albumin 2.2 g/dL, C-reactive protein 6.0 mg/dL. Enhanced computed tomography (CT) revealed mediastinal abscess with free air and atelectasis of the right lower lobe. Endoscopy and upper gastrointestinal series revealed perforation on the right wall of the middle thoracic esophagus 32–35 cm distant from the incisors and blood clots in stomach (Fig. 2).

The next day, esophageal bypass surgery was performed. A gastric conduit from the greater curvature was formed for reconstruction. The cervical esophagus was divided above the sternal notch. The gastric conduit was pulled up through the retrosternal tunnel and anastomosed to the cervical esophagus. The drainage tube was retrograde into the esophagus through the divided stomach as a tube esophagostomy.

**Fig. 3:**
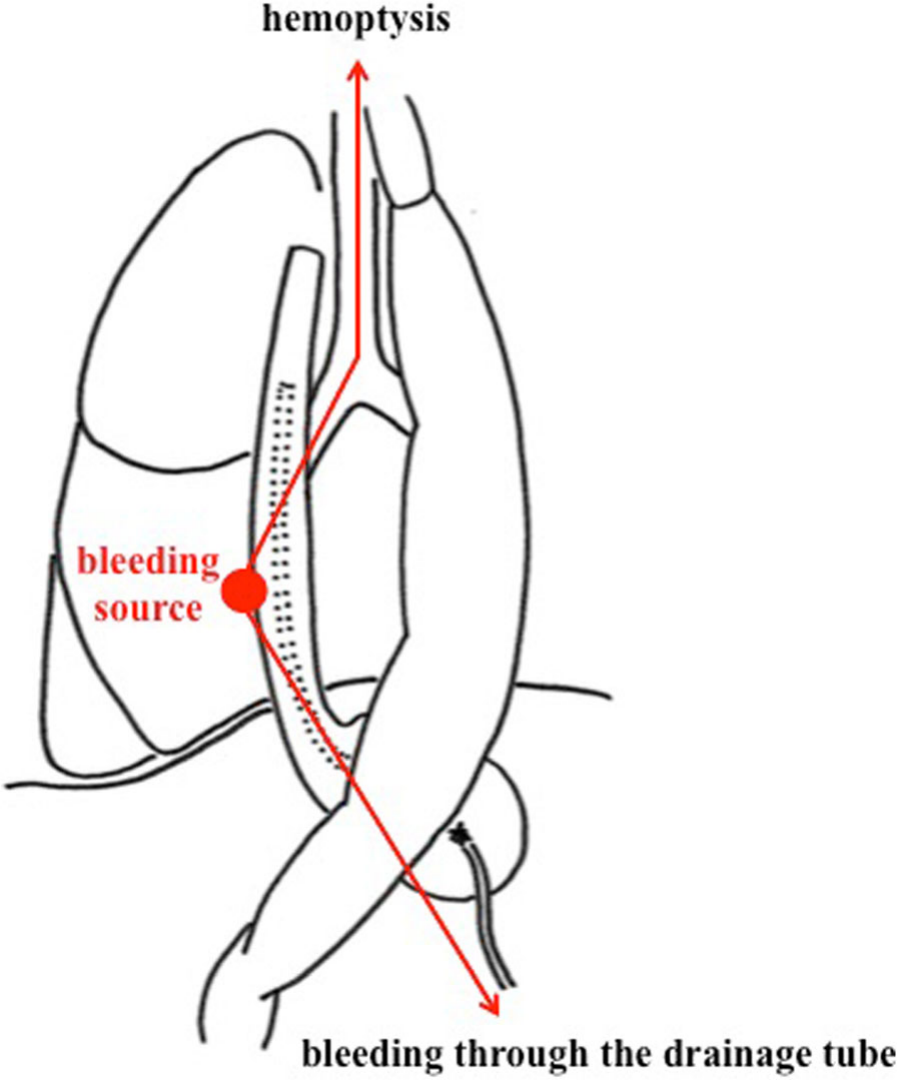
The patient developed hemoptysis concomitantly with bleeding through the drainage tube inserted into the esophagus

**Fig. 4:**
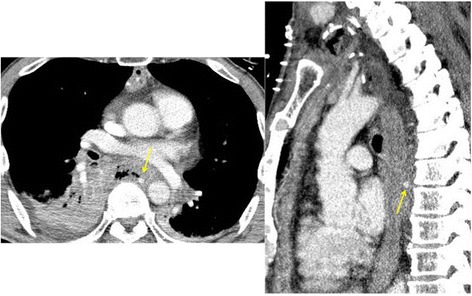
Enhanced computed tomography on hemoptysis revealed contrast medium extravasation, indicating a pseudoaneurysm (*arrow*) at the mediastinum

**Fig. 5:**
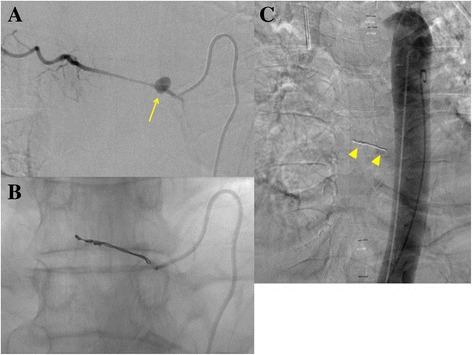
**a** A pseudoaneurysm of the right seventh intercostal artery (*arrow*) was confirmed by digital subtraction angiography at the same location as on enhanced computed tomography. **b** After embolization. **c** Aortography revealed the positional relationship between the microcoils (*arrowhead*) and the descending aorta

**Fig. 6:**
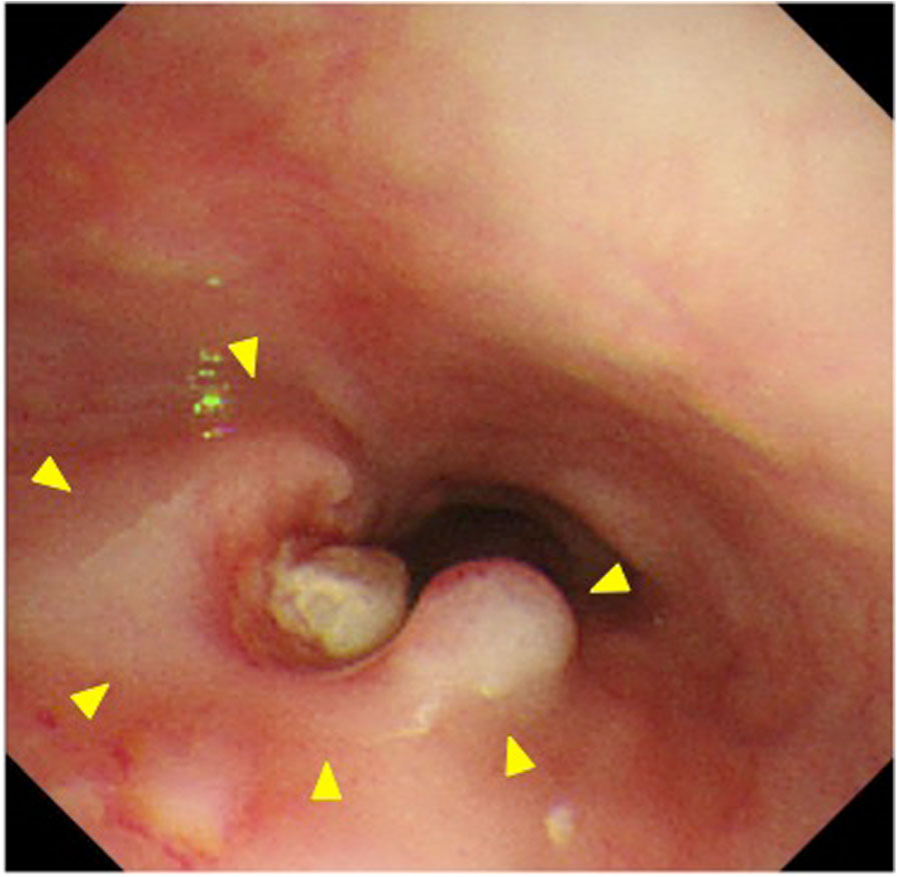
Retrograde endoscopy through the route of the drainage tube inserted into the esophagus revealed a protruded tumor (*arrowhead*) at the right and posterior wall of the middle thoracic esophagus

Three days later, he suddenly developed hemoptysis through the endotracheal tube. Emergency bronchoscopy revealed massive bleeding from the bronchus of the right lower lobe. At the same time, he presented with similar bleeding through the drainage tube inserted into the esophagus (Fig. 3). Enhanced CT revealed contrast medium extravasation, indicating a pseudoaneurysm at the mediastinum (Fig. 4). As angiography showed a pseudoaneurysm of the right seventh intercostal artery, embolization was performed with microcoils (0.018 inch, 3 × 2 mm, Tornado® Embolization Microcoil Platinum; Cook Bloomington, IN, USA) (Fig. 5). The patient recovered uneventfully and was discharged from our hospital on postoperative Day 32. Before discharge, endoscopy through the route of the drainage tube showed a protruded tumor at the middle thoracic esophagus (Fig. 6). A biopsy of the tumor revealed residual squamous cell carcinoma of the esophagus.

Postoperatively the patient received chemotherapy. He underwent esophagectomy concomitantly with right lower lobectomy 5 months after esophageal bypass. The procedure was performed through right posterolateral thoracotomy. After esophagectomy, an embolized coil was detected at the posterior chest wall. A histopathological examination revealed that the residual squamous cell carcinoma of the esophagus had directly invaded segment 6 of the right lung. Thereafter, he was diagnosed with systemic metastasis and died 5 months after esophagectomy.

## Discussion

The arterial blood supply of the thoracic esophagus comes from the bronchial arteries, the esophageal arteries, and the right third or fourth intercostal arteries [2]. We performed a search of PubMed using the following key words: “esophageal cancer/bleeding.” Only four cases of non-aortic AEF for thoracic esophageal cancer have been reported in the published literature to date. While the bronchial artery has the most commonly been reported to be the responsible artery of non-aortic AEF, there has only been one published report of intercostal AEF (Table 1).

**Table 1:** Reported cases of non-aortic arterio-esophageal fistula

No.	Author	Year	Age	Sex	Stage	Location	Responsible artery	RT or CRT	Treatment	AEF treatment
1	Taniguchi [4]	2011	74	M	T4N-M-	Mt	Bronchial artery	CRT	TAE	Success
2	Taniguchi [4]	2011	65	M	T4N-M-	Ut	Intercostal artery	CRT	TAE	Success
3	Taniguchi [4]	2011	56	M	T4N-M-	Mt	Bronchial artery	CRT	TAE	Success
4	Aoki [8]	2016	66	M	T3N1M1	-	Right bronchial artery	RT	TAE	Success
5	Our case	2017	69	M	T3N1M0	Mt	Right seventh intercostal artery	CRT	TAE	Success

*M* male, *Ut* upper thoracic esophagus, *Mt* middle thoracic esophagus, *RT* radiotherapy, *CRT* chemoradiotherapy, *TAE* transcatheter arterial embolization, *AEF* arterio-esophageal fistula, *hyphen* (-) not described

The intercostal arteries can form a pseudoaneurysm due to iatrogenic injury and trauma [3], but they are extremely rare as a source of esophageal bleeding. Taniguchi et al. reported two cases of bronchial AEF and one case of intercostal AEF [4]. They all underwent CRT for T4 esophageal cancer with clinical invasion of the aorta. CRT can induce fistula formation by damaging the walls of the esophagus and adjacent organs [1]. Although the present case had T3 esophageal cancer at the initial diagnosis, CRT and tumor regrowth may have contributed to an increased risk of fistula formation. In addition, the inflammation of a mediastinal abscess along the chest wall may have resulted in intercostal AEF.

More than half of lethal bleeding events in patients with radiated esophageal cancer are hematemesis due to aortic AEF [5]. In the present case, enhanced CT on admission revealed that the mediastinal abscess mainly spread around the right side of the esophagus with no evidence of aortic AEF. We considered his hematemesis to be temporary bleeding from the main tumor because his symptom was vomiting blood and the source of bleeding was not detected on endoscopy or enhanced CT. As the esophago-mediastinal fistula caused by esophageal perforation formed a further fistula to the intercostal artery and lung, the present patient developed hemoptysis. The occurrence of hemoptysis after esophageal bypass prompted us to consider a diagnosis of intercostal AEF. When patients with locally advanced esophageal cancer present with hematemesis and/or hemoptysis, the possibility of non-aortic AEF should be considered.

TAE has become the first-line therapy for the management of acute nonvariceal upper gastrointestinal bleeding which is resistant to endoscopic treatment [6]. TAE was also effective for intercostal AEF in terms of the accuracy and rapidity of hemostasis. Esophageal bypass for patients with esophago-respiratory fistula makes it possible to divide the respiratory and alimentary tracts, which can prevent pulmonary sepsis caused by the continuous supply of saliva, digestive juice, and/or necrotic tissue from the tumor [7]. In the present case, esophageal bypass was performed to control mediastinitis and allow for the resumption of oral intake by dividing the focus of infection and the alimentary tracts under the same concept as that reported by Nakajima et al. [7]. The feasible and radical treatment for intercostal AEF was esophageal bypass because mediastinitis was one of the main causes of intercostal AEF. Due to successful TAE and esophageal bypass, the present case did not suffer from rebleeding after TAE.

## Conclusions

When patients with thoracic esophageal cancer, especially those with a history of radiotherapy and/or mediastinitis, present with hematemesis and/or hemoptysis, the possibility of non-aortic AEF should be considered. TAE is an effective treatment for non-aortic AEF.

## Abbreviations

AEF: Arterio-esophageal fistula
CRT: Chemoradiotherapy
CT: Computed tomography
TAE: Transcatheter arterial embolization
